# A Comparison of Full Arch Trueness and Precision of Nine Intra-Oral Digital Scanners and Four Lab Digital Scanners

**DOI:** 10.3390/dj9070075

**Published:** 2021-06-23

**Authors:** Adam B. Nulty

**Affiliations:** School of Dentistry, University of Leeds, Woodhouse Lane, Leeds LS2 9JT, UK; dnabn@leeds.ac.uk

**Keywords:** intra-oral scanners, digital dentistry, trueness, precision, lab scanners

## Abstract

(1) Background: The purpose of this study is to evaluate the full arch scan accuracy (precision and trueness) of nine digital intra-oral scanners and four lab scanners. Previous studies have compared the accuracy of some intra-oral scanners, but as this is a field of quickly developing technologies, a more up-to-date study was needed to assess the capabilities of currently available models. (2) Methods: The present in vitro study compared nine different intra-oral scanners (Omnicam 4.6; Omnicam 5.1; Primescan; CS 3600; Trios 3; Trios 4; Runyes; i500; and DL206) as well as four lab light scanners (Einscan SE; 300e; E2; and Ineos X5) to investigate the accuracy of each scanner by examining the overall trueness and precision. Ten aligned and cut scans from each of the intra-oral and lab scanners in the in vitro study were brought into CloudCompare. A comparison was made with the master STL using the CloudCompare 3D analysis best-fit algorithm. The results were recorded along with individual standard deviation and a colorimetric map of the deviation across the surface of the STL mesh; a comparison was made to the master STL, quantified at specific points. (3) Results: In the present study, the Primescan had the best overall trueness (17.3 ± 4.9), followed by (in order of increasing deviation) the Trios 4 (20.8 ± 6.2), i500 (25.2 ± 7.3), CS3600 (26.9 ± 15.9), Trios 3 (27.7 ± 6.8), Runyes (47.2 ± 5.4), Omnicam 5.1 (55.1 ± 9.5), Omnicam 4.6 (57.5 ± 3.2), and Launca DL206 (58.5 ± 22.0). Regarding the lab light scanners, the Ineos X5 had the best overall trueness with (0.0 ± 1.9), followed by (in order of increasing deviation) the 3Shape E2 (3.6 ± 2.2), Up3D 300E (12.8 ± 2.7), and Einscan SE (14.9 ± 9.5). (4) Conclusions: This study confirms that all current generations of intra-oral digital scanners can capture a reliable, reproducible full arch scan in dentate patients. Out of the intra-oral scanners tested, no scanner produced results significantly similar in trueness to the Ineos X5. However, the Primescan was the only one to be statistically of a similar level of trueness to the 3Shape E2 lab scanner. All scanners in the study had mean trueness of under 60-micron deviation. While this study can compare the scanning accuracy of this sample in a dentate arch, the scanning of a fully edentulous arch is more challenging. The accuracy of these scanners in edentulous cases should be examined in further studies.

## 1. Introduction

The emergence and use of intra-oral scanners in dental clinics has provided a better experience for the patient and an easier way of creating an impression model in a more predictable and repeatable way to alleviate problems or complications encountered in a conventional workflow using traditional methods with a tray-based impression [1]. When the digital intra-oral scanners were introduced in the 1980s, the fully digital workflow became a reality.

Several recent technical improvements have made the intra-oral digital scanner a central part of modern dental surgery, enabling same-day dentistry, reducing the need for conventional impressions, or even replacing them entirely. Many clinicians are now starting to use a digital scanner, and there is a number of well-performing scanners on the market.

There are many clinical advantages compared to conventional impression taking, namely speed, patient comfort, efficacy, and several new ways dentists can predictably work once the intra-oral situation is digitised. Also, a significant factor is that the use of a digital scanner can reduce costs in the long run [2,3,4].

An alternative way of digitising the intra-oral environment is through the capture of either an impression or casted model in a lab scanner, but this study will focus on the use of direct intra-oral scanners and compare their relative accuracy to a base-level lab scanner.

While there have been several previous studies comparing the accuracy of intra-oral scanners, there are no currently published studies that compare the scanners available in 2020, namely those in the present study [5,6,7,8,9], which can therefore be seen as a gap in the literature that must be studied. It has been suggested that using a digital intra-oral scanner for a full arch scan is less acceptable, as the longer scan distance may introduce a possible error [10].

The technology used in scanners varies, and therefore the ease of use, efficacy, and accuracy in terms of trueness and precision may vary. The scanner will measure a number of intra-oral readings and create a three-dimensional image using a mathematical algorithm. Due to the limited field of view of an intra-oral scanner, the single-point cloud map generated with each scan frame cannot cover all of the tooth surfaces. Thus, the scanner software overlaps these frames with subsequently captured frames to create a unified, 3D mesh representing the full arch [11]. Depending on the manufacturer and the scanning technology employed, various algorithms and stitching methods combine these individual images. However, these methods inherently contain a degree of error that can accumulate across the dental arch when a full arch scan is performed [12,13,14]. The outcome of the digital models is based on how reproducible and accurate the scan is. The varying degree to which the scanners perform this stitching function will mean that the choice of the scanner may influence the overall accuracy of the resulting scan [15,16].

This study focuses on the precision and trueness of nine modern intra-oral digital scanners and four digital lab scanners. Accuracy consists of trueness and precision (ISO 5725-1) [17]. Trueness is, by definition, an indication of how similar a measurement is to a known measured value [18]. In the present study, trueness describes the deviation of the measurements in the data set compared to the actual dimensions of the scanned object. Therefore, high trueness indicates that the scanner delivers a result that is very close to the actual dimensions of the object being scanned [18]. Precision expresses the degree of reproducibility or agreement between repeated measurements [18]. In the present study, precision describes how close each measurement in the data set is to the other measurements taken by the same scanner. Therefore, higher precision means that a scanner is capable of taking consistently repeatable scans. As the scan data obtained in a clinical situation are the basis of the planning and design, it is of great importance that the scan is recording an accurate reading in a reproducible way. Therefore, we compared the trueness and precision to get a value of accuracy on scanning a master model for the leading scanners currently in use and two lab scanners for comparison.

The null hypothesis was that no differences would be found between the various scanners regarding trueness and precision.

A secondary null hypothesis was that there would be no difference between the lab scanners and intra-oral scanners regarding trueness and precision.

## 2. Materials and Methods

### 2.1. Study Model

**Figure 1:**
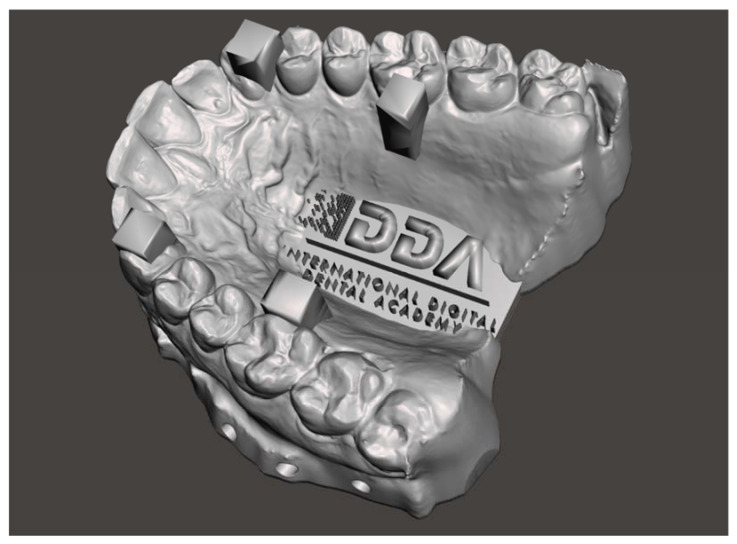
The IDDA Calibration Model. This master model was printed using an Asiga Max UV and NextDent Model Resin at 50-micron layer height. This printer and resin combination was chosen for high precision and low reflectivity to facilitate the acquisition with the intra-oral scanners used in the study [20].

The present study used the International Digital Dental Academy [19] Calibration Model (Figure 1), which represents three different situations in the maxilla:

- a fully dentate arch;
- four regular structures in the form of columns of known width and separation; and
- a high degree of surface morphology.

### 2.2. Scanners in the Study

The scanners used in the present in vitro study are summarised in Table 1.

### 2.3. Design of the Study

The present in vitro study compared nine different intra-oral scanners (Omnicam with 4.6 Software, Omnicam with 5.1 Software, and Primescan—Dentsply Sirona, York, PA, USA; CS 3600—Carestream Dental, Atlanta, GA, USA; Trios 3 and Trios 4—3Shape, Copenhagen, Denmark; Runyes Quickscan—Ningbo Runyes Medical Instrument Co., China; i500—Medit, Seongbuk-gu, Seoul, Korea; and DL206—Guangdong Launca Medical Device Technology Co., Ltd., Dongguan, China) as well as four lab light scanners (Einscan SE—Shining 3D, Hangzhou, Zhejiang, China; UP3D 300e—Shenzhen UP3D Tech Co., Ltd., Shenzhen, China; E2—3Shape, Copenhagen, Denmark; and Ineos X5—Dentsply Sirona, York, PA, USA) to investigate the accuracy of each scanner by examining the overall trueness and precision.

The master model was acquired for each of the above scanners and compared with the Ineos X5 Lab Scanner. This structured light lab scanner is accredited to be accurate within 2.1 microns (ISO 12836) [21]. A sample size of 10 for each scanner was determined by using a sample size calculation with 95% confidence level and a margin of error of 5%. This has been confirmed by several authors to be acceptable to obtain statistically significant results [13,22,23].

A single operator, an expert in digital dentistry familiar in use and experienced with multiple manufacturers of scanners, then began to scan the master model using each of the scanners available, capturing ten scans in total for each scanner. To avoid operator fatigue, the sequence of scans was randomised with intervals between each scan. The scanner used for each scan was also randomised to prevent bias.

**Figure 2:**
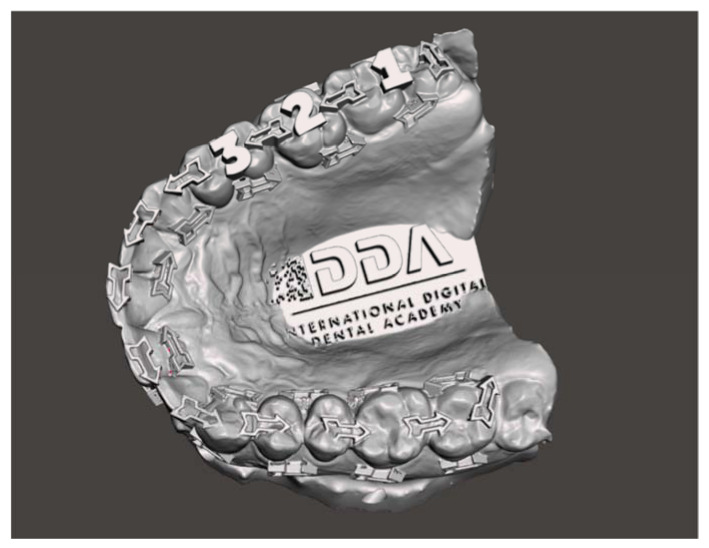
The IDDA Scan Method Training Model.

In each scan, the method of scanning followed the International Digital Dental Academy Scan Training Model [19] (Figure 2): starting on the upper left-most distal molar, continuing occlusally across the full arch, pivoting to the palatal side to capture the palatal surfaces, and then returning along the buccal surface with a constant progression.

This scanning method captures a little of the palatal and buccal surface when scanning the occlusal arc, capturing the areas of interest of each surface while maintaining a common framework for the meshes to align. The scans were exported as an STL format file using the manufacturer’s proprietary and recommended conversion pathway.

The scans were then imported into Meshlab (ISTI-CNR, Pisa, Italy) [24] (an open-source system for processing and editing 3D triangular meshes) and aligned.

This method was repeated with each of the intra-oral scanners and lab scanners in the study. Once all of the scans were aligned, the surface meshes were digitally cut using a template and exported to give ten resulting meshes for each scanner to be used to compare the trueness and precision evaluations.

### 2.4. Evaluating Trueness

For trueness, the master model scans using the Ineos X5 were used as a baseline measurement against the Original STL of the IDDA Calibration Model. Each of the ten aligned and cut scans from each of the scanners in the in vitro study was brought into CloudCompare (an open 3D point cloud and mesh processing and comparison software), where the scans were further aligned and calibrated using the fine alignment algorithm. Each data set was then compared with the master STL using the CloudCompare 3D analysis best-fit algorithm. Trueness was defined as the mean deviation value for the superimposition of each scan. The results were recorded along with the standard deviation for each scan.

### 2.5. D Deviation

The CloudCompare software allows the generation of a colorimetric map of the deviation across the surface of the STL mesh as compared to the master STL, quantified at specific points. The colour map indicates deviation inward (blue) or outward (red), while green indicates minimal deviation. The same C2M colour deviation scale was employed to illustrate the minimum and maximum deviations for each comparison. The colour scale ranged from a maximum and minimum deviation of +200 (outward/red) and −200 μm (inward/blue).

### 2.6. Evaluating Precision

All possible pairwise comparisons were made using a one-way analysis of variance (ANOVA) for independent groups, with a Tukey significance level of 0.05, of multiple comparisons using SPSS 26 by IBM [25]. Bartlett’s test was used to test the homogeneity of variances. Precision was defined from the superimposition between the different scans made with the same intra-oral scanner. The comparisons available for each scanner were calculated and the precision of each scanner was then expressed as a mean.

### 2.7. Surface Detail Observational Comparison

Finally, an illustration of the surface features was made by capturing the wireframe of the premolar/molar region incorporating the calibration column.

## 3. Results

**Figure 3:**
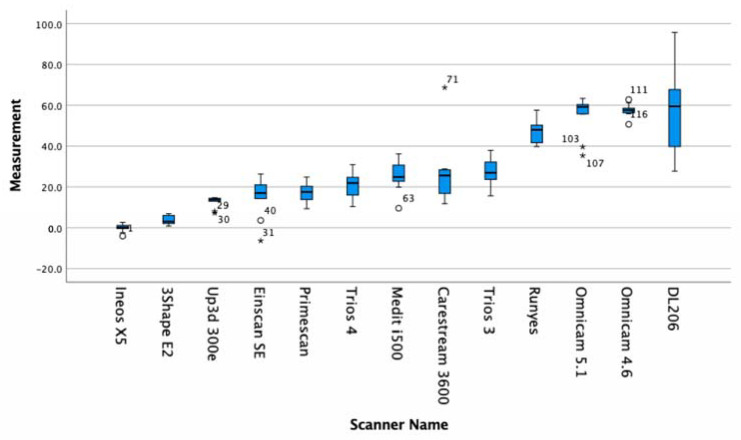
Box plot of each data set for each scanner in the present study. * represents outlier.

**Figure 4:**
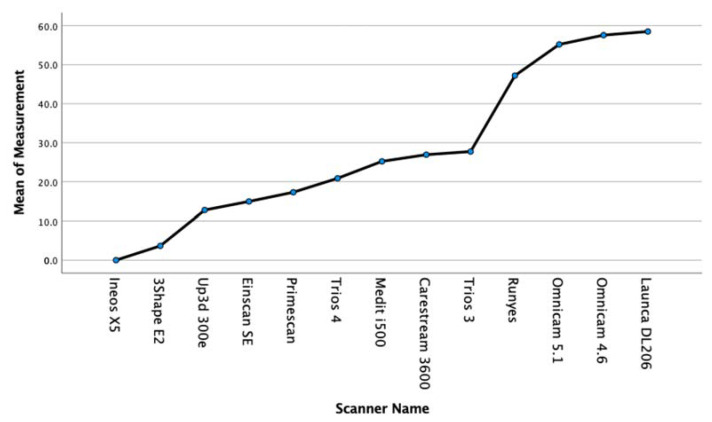
Means plot of precision for each scanner.

Trueness and Precision results are summarised in Table 2 and Table 3 and in Figure 3 and Figure 4. Table 4 shows the Anova significance between groups.

In the present study, the Primescan had the best overall trueness (17.3 ± 4.9), followed by (in order of increasing deviation) the Trios 4 (20.8 ± 6.2), i500 (25.2 ± 7.3), CS3600 (26.9 ± 15.9), Trios 3 (27.7 ± 6.8), Runyes (47.2 ± 5.4), Omnicam 5.1 (55.1 ± 9.5), Omnicam 4.6 (57.5 ± 3.2), and Launca DL206 (58.5 ± 22.0). We can see that the scanners that employ the confocal microscopy technology have the highest levels of trueness.

With regards to the lab light scanners, the Ineos X5 had the best overall trueness with (0.0 ± 1.9), followed by (in order of increasing deviation) the 3Shape E2 (3.6 ± 2.2), Up3D 300E (12.8 ± 2.7), and Einscan SE (14.9 ± 9.5)

The precision results are summarised in Table 3. In brief, the Ineos X5 was statistically more precise than all of the intra-oral scanners. The Primescan intra-oral scanner was the only intra-oral scanner statistically grouped in precision with desktop lab scanners E2 and 300E. Six of the intra-oral scanners, the Primescan, Trios 4, i500, 3600, and Trios 3, were statistically more precise than the Runyes, Omnicam, and DL206.

**Figure 5:**
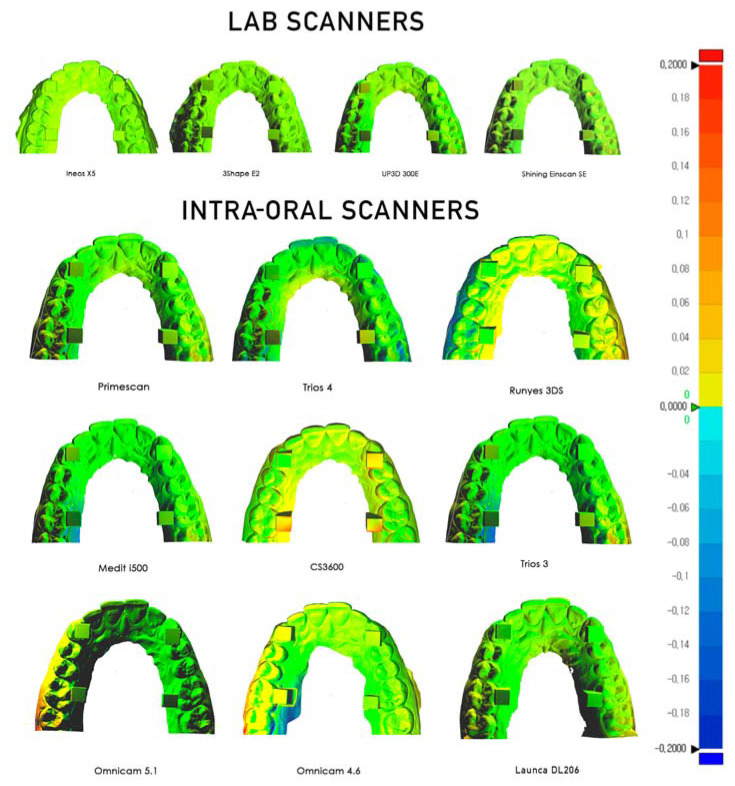
Colorimetric map of the deviation.

**Figure 6:**
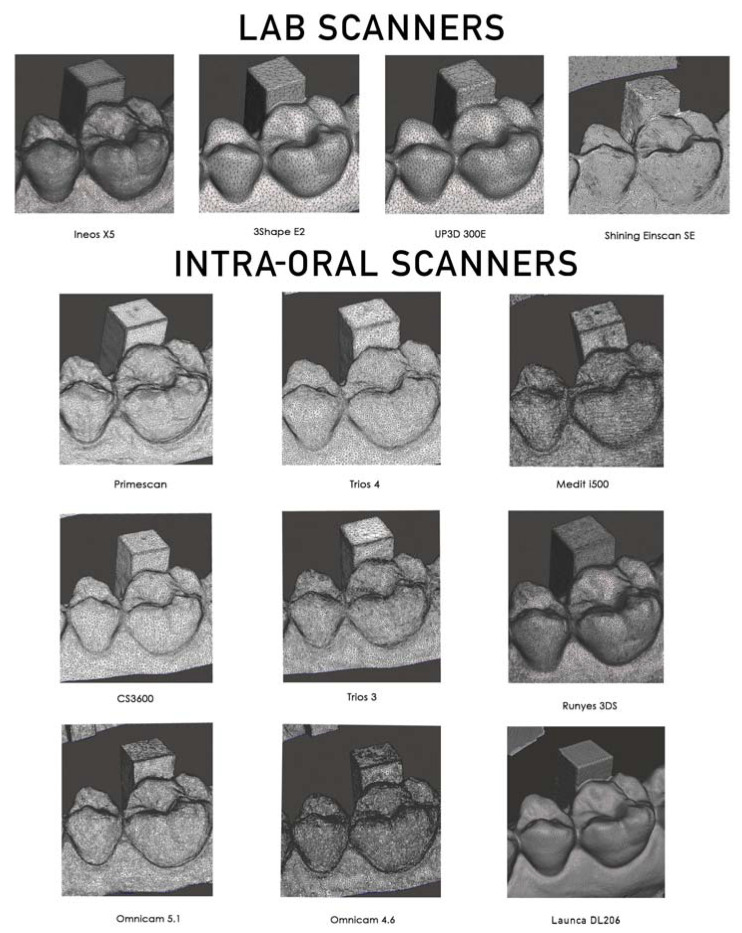
Comparison of triangular meshes.

All intra-oral scanners present a mean error below 60 microns across a full arch comparison. (Figure 5). Five current-generation scanners in the study (excluding the Runyes, Omnicam, and Launca) provide a mean error below 30 microns with a low deviation, which confirms a high level of reliability of the Primescan, Trios 3 and 4, i500, and CS3600. The oldest model of scanner, the Omnicam, was tested with two varieties, running software version 4.6 and version 5.1. The current 5.1 hardware and software is credited as having improved the accuracy of this scanner that has been on the market for over eight years. Our results show that while the mean deviations were higher than that of the other scanners, the later software version improved both the mean accuracy and lowered the standard deviation. Interestingly, the Einscan SE lab scanner produced results with a high degree of trueness (15.6 ± 9.5). However, this was overall trueness to the master STL, and on observational inspection of the triangular meshes, it is evident that the surface detail is lacking (Figure 6).

## 4. Discussion

The null hypothesis was rejected in that significant differences were found among some of the digital intra-oral scanners and lab scanners regarding trueness and precision.

With regards to the secondary null hypothesis that there would be no difference between the lab scanners and intra-oral scanners regarding trueness and precision, this was partially rejected, as one intra-oral scanner, the Primescan, whilst having a statistically significant difference to the Ineos X5 lab scanner, proved the secondary null hypothesis correct in terms of comparison to the other lab scanners.

The evolution of intra-oral scanners to lead to one performing at a statistically significant level in comparison to lab scanners is remarkable. Whilst clear differences between the scanners were found, the performance of these scanners can be seen to be exceptional with all lab and intra-oral scanners performing with overall trueness under 60 μm. The emergence of intra-oral scanners has intended to provide a better experience for the patient and also an easier way of creating a model in a more predictable and repeatable way to alleviate problems/complications encountered in conventional methods/impressions [1].

As digital intra-oral scanners are becoming more prevalent in practice, it has allowed us to provide same-day dentistry in a predictable and efficient way. This has led to the advent of same-day dentistry where indirect restorations can be placed in the same visit. There has, however, been a great deal of discussion around the accuracy and reproducibility of digital intra-oral scanners versus the conventional analogue techniques—e.g., digital vs. analog impression [26,27,28]. It is generally accepted that the marginal fit of complete coverage restorations constructed using digital scanners show higher accuracy than conventional impressions, but full arch scans are more controversial and technique-sensitive, and the scanner being used plays a big part in the overall accuracy and precision of the digital model created [29,30].

The purpose of this study is to address these issues around precision/trueness and accuracy for a full arch scan. We have studied these parameters for seven digital intra-oral scanners and four lab scanners. This is the most up-to-date study on the most recent scanners that have been released as of the start of 2021. However, this study did not replicate an actual clinical situation and has several limitations. In most patients, multiple surfaces and materials are scanned, including various restorative materials, dentin, enamel, and soft tissues. Inherent anatomy-related changes in arch shape or jaw opening mean that this in vitro study is fundamentally limited, and in vivo studies using these scanners would be important to further illustrate the differences in accuracy. Further studies should be completed to determine whether these factors may affect full arch accuracy in these current generation scanners.

In the present study, only one clinician performed the scans on the master model to produce the data set for each scanner. This is important as variation in scan strategy can affect the accuracy of stitching, which in turn would impact the significance of the results comparison [31,32,33].

The terms trueness and precision have been prescribed in ISO 5725-1 to represent the accuracy of the measurement method to evaluate digital intra-oral scanners [18]. Lab scanners are known to be more accurate, as they use lasers or structured light and are not purely optical, with a limited field of view, such as digital intra-oral scanners, and also exhibit fewer inhibiting factors (for example, lens wetting, reflections from scanned surfaces, movement of the tongue/soft tissues, etc.) to deal with when scanning [34] and have therefore been used in this study as the benchmark for the accuracy and precision of the scanners.

There are many published studies that compare the accuracy of digital intra-oral scanners [22,23,24,25,26,27]—they compare different generations of scanners and do not necessarily compare new technology and software updates for the older technology scanners—e.g., the Omnicam with 4.6 software compared to the Omnicam running 5.1 software. Mathematical and software developments of the stitching algorithm [35,36] have improved, and this is clear in the results of this study, where the later software version combined with more recent computer hardware resulted in a more accurate data set. It has also been shown that calibration plays a very big part in the accuracy and precision of the scanner [37], and in the present study, all scanners were calibrated immediately prior to the capture of the scans in each data set.

A number of limitations are suggested in our in vitro study, namely the in vivo complications, such as saliva, blood, patient interaction, etc. These need to be accounted for and may impact the results in an in vivo patient setting.

Looking at the observational comparison of the triangular meshes, it becomes apparent that there are clear differences in the ability of these lab and intra-oral scanners to accurately portray surface features and marginal integrity. In terms of the lab scanners, we can see that, as the deviation in trueness increases, so does the lack of detail. However, this is not the case for the intra-oral scanners. There is a large variation in the portrayal of occlusal anatomy as well as some degree of difficulty for some of the scanners to efficiently process flatter areas. Whilst it may seem appropriate to look at the total triangle count for the scans, each scanner processes the point clouds differently, converting the point cloud created during scanning to a useable CAD triangle mesh. The more well-known brand scanners from Dentsply Sirona, 3Shape, and Carestream show obvious variation in the triangular mesh size and density, whilst the newer scanners from Medit, Runyes, and Launca are very regular in their mesh density. This may be because these scanners have a longer history of research and development, and as such, the algorithms employed to convert the point clouds recorded into triangular meshes will have had more time to be optimised. One of the most impressive meshes on first observation was the Launca DL206 scan. This scanner was just released at of the start of 2021, and whilst the trueness is on par with the Runyes and Omnicam scanners, the triangular mesh of this scanner shows an impressive level of detail. However, without a full understanding of the manufacturer’s particular patented methods of algorithmic conversion from point cloud to triangular mesh, this is a potential limitation of comparing the appearance of triangular meshes and total triangle count.

The fast-paced changes and developments in modern dentistry within CAD/CAM, digital impression registration, and chair-side production are remarkable and likely to quickly become an even greater factor in developing modern dentistry. A central part of the modern digital dentistry office is registering a true and accurate scan of the intra-oral anatomy. The use of digital intra-oral scanners is well established, and a number of well tested scanners are available on the market. Needless to say, it is a competitive field for the manufacturers of dental equipment, and we can look forward to ongoing improvements. It is widely accepted that the use of a digital intra-oral scanners enhances the patient experience. The in-house workflow gives the clinician opportunities to capture a detailed, three-dimensional picture of the intra-oral situation, thus enabling same-day dentistry and many new opportunities.

An abundance of data indicate that, although we can very accurately record the situation and produce reliable digital models of preparations, we have limited data of trueness and accuracy across the variety of devices commercially available. Some studies suggest that scanners can replace impressions for dental preparations, but it is not clear if they can replace a conventional impression in every situation [20,21,22,23,38,39,40].

Several recent studies have shown that digital intra-oral scanners are accurate, but some variations are noted. The older studies suggesting that accuracy of scanners is limited and suggest using scanners for smaller prosthetic situations seem to be based on limited numbers of scanners and notably older scanners [41]. The present study includes the latest scanners and shows a very different situation, as the majority of current scanners with the latest software produced results that were accurate to within 30 microns.

## 5. Conclusions

At the time of completing the present study, there have been very few studies comparing the accuracy of the various current intra-oral scanners to assess full arch accuracy.

Our present study aimed to compare the full arch trueness and precision of the leading intra-oral scanners available in 2020 (specifically the Dentsply Sirona Primescan and Omnicam (both 4.6 and 5.1 version), 3Shape Trios 3 and 4, Carestream 3600, Launca DL206, Runyes, and Medit i500) as well as a low-cost lab light scanner (Shining Einscan SE) and more mainstream dental lab scanners (Dentsply Sirona Ineos X5, 3Shape E2, and UP3D 300e).

Each scanner took ten scans, and all data sets were compared using Cloudcompare to evaluate the trueness and precision. The study results showed that the Primescan produced a very low amount of overall deviation and recorded the most accurate results, which were statistically similar to all lab scanners except the Ineous X5. The Primescan was followed closely by the Trios 4, Medit i500, CS3600, and Trios 3 as the second most accurate data set of intra-oral scanners with no statistical difference between the overall results of the current range of scanners: Primescan, Trios 3 and 4, i500, and 3600. The results confirmed a statistical difference between these scanners and the previous generation scanner, the Omnicam, and the Runyes and Launca DL206. However, the later generation hardware and software version of the Omnicam did produce more accurate results, and these results of these three scanners were still within an acceptable range for clinical usefulness.

While this and other studies have looked at the accuracy of these scanners, an interesting observational outcome of the present study was examining the close-up anatomical detail shown by the triangular meshes. There is a very clear and noticeable difference between the level of detail shown by the Ineos X5 and the other lab scanners and similarly with the Primescan. The scanners show a variation in their ability to efficiently portray the flat surfaces while also showing higher concentration in triangular mesh around important surface features and angles. The two newer scanners, the Runyes and particularly the Launca DL206, show an impressive level of detail, with the Launca DL206 scanner mesh being evenly rendered with a very dense mesh. This is noticeable in the Launca DL206′s STL file size being larger than all other scanners.

This study confirms that all of the intra-oral digital scanners can capture a reliable, reproducible full arch scan in dentate patients. However, the scanning of an edentulous full arch is more challenging and deserves further investigation.

Following this study, further research is needed on these scanners in various settings, and the evidence must be confirmed in a clinical setting.

## Figures and Tables

**Table 1:** The digital scanners used in this study.

Name	Manufacturer	Technology	STL Export	PLY/OBJ Colour Export
Omnicam 4.6	Dentsply Sirona, York, PA, USA	Structured light—Optical triangulation and confocal microscopy	YES	NO
Omnicam 5.1	Dentsply Sirona, York, PA, USA	Structured light—Optical triangulation and confocal microscopy	YES	NO
Primescan	Dentsply Sirona, York, PA, USA	Structured light—Confocal microscopy with Smart Pixel sensor.	YES	NO
CS3600	Carestream Dental, Atlanta, GA, USA	Structured LED light—Active Speed 3D Video™	YES	YES
Trios 3	3-Shape, Copenhagen, Denmark	Structured light—Confocal microscopy and Ultrafast Optical Scanning™	YES	YES
Trios 4	3-Shape, Copenhagen, Denmark	Structured light—Confocal microscopy and Ultrafast Optical Scanning™	YES	YES
Runyes	Ningbo Runyes Medical Instrument Co., Shenzhen, China	Structured light—Active Speed 3D Video™	YES	YES
Launca DL206	Guangdong Launca Medical Device Technology Co., Ltd., Dongguan, China	Structured light—Active Speed 3D Video™	YES	YES
I500	Medit, Seongbuk-gu, Seoul, Korea	Structured light—Active Speed 3D Video™	YES	YES
Einscan SE	Shining 3D, Hangzhou, Zhejiang, China	Optical Blue Structured Light	YES	NO
UP3D 300E	Shenzhen UP3D Tech Co., Ltd., Shenzhen, China	Optical Blue Structured Light	YES	NO
E2	3-Shape, Copenhagen, Denmark	Optical Blue Structured Light	YES	NO
Ineos X5	Dentsply Sirona, York, PA, USA	Optical Blue Structured Light	YES	NO

**Table 2:** Mean trueness and standard deviation of each scanner in comparison to the master scan from the Ineos X5 in order of ascending mean deviation and the significance compared to the Ineos X5 results.

Name	Mean (μm)	Std. Deviation (μm)	*p* Value
Ineos X5	0.0	1.9	1.000
3Shape E2	3.6	2.2	0.125
UP3D 300E	12.8	2.7	0.029
Einscan SE	14.9	9.5	0.004
Primescan	17.3	4.9	<000.1
Trios 4	20.8	6.2	<000.1
Medit i500	25.2	7.3	<000.1
CS3600	26.9	15.9	<000.1
Trios 3	27.7	6.8	<000.1
Runyes	47.2	5.4	<000.1
Omnicam 5.1	55.1	9.5	<000.1
Omnicam 4.6	57.5	3.2	<000.1
Launca DL206	58.5	22.0	<000.1

**Table 3:** Tukey homogenous subsets of compared means (subset for alpha = 0.05).

Name	1	2	3	4	5
Ineos X5	0.000				
3Shape E2	3.7	3.7			
UP3D 300E	12.8	12.8	12.8		
Einscan SE		15.0	15.0	15.0	
Primescan		17.3	17.3	17.3	
Trios 4			20.9	20.9	
Medit i500			25.2	25.2	
CS3600			26.9	26.9	
Trios 3				27.7	
Runyes					47.2
Omnicam 5.1					55.2
Omnicam 4.6					57.6
Launca DL206					58.5
*p* Value (Sig)	0.125	0.072	0.051	0.123	0.271

**Table 4:** Anova sig. between groups.

Anova					
Measurement	Sum of Squares	Df	Mean Square	F	Sig.
Between Groups	28,324.784639	8	3540.598080	29.235153	0.000
Within Groups	9809.712588	81	121.107563		
Total	38,134.497226	89			

## Data Availability

The datasets generated during and/or analysed during the current study are available from the corresponding author on reasonable request.
